# Toward the Autism Motor Signature: Gesture patterns during smart tablet gameplay identify children with autism

**DOI:** 10.1038/srep31107

**Published:** 2016-08-24

**Authors:** Anna Anzulewicz, Krzysztof Sobota, Jonathan T. Delafield-Butt

**Affiliations:** 1 Jan Matejko Academy of Fine Arts, Kraków, Poland; Jagiellonian University, Krakow, Poland; 2 Harimata Sp. z.o.o., Kraków, Poland; 3 University of Strathclyde, Glasgow, UK

## Abstract

Autism is a developmental disorder evident from infancy. Yet, its clinical identification requires expert diagnostic training. New evidence indicates disruption to motor timing and integration may underpin the disorder, providing a potential new computational marker for its early identification. In this study, we employed smart tablet computers with touch-sensitive screens and embedded inertial movement sensors to record the movement kinematics and gesture forces made by 37 children 3–6 years old with autism and 45 age- and gender-matched children developing typically. Machine learning analysis of the children’s motor patterns identified autism with up to 93% accuracy. Analysis revealed these patterns consisted of greater forces at contact and with a different distribution of forces within a gesture, and gesture kinematics were faster and larger, with more distal use of space. These data support the notion disruption to movement is core feature of autism, and demonstrate autism can be computationally assessed by fun, smart device gameplay.

Autism spectrum disorder (ASD) is a childhood neurodevelopmental disorder. Its global prevalence is estimated at 1 in 160 children^1^. The European and North American prevalence of autism is estimated to be 1 in 68 children^2^. In the UK, *ca.* 700,000 individuals live with autism^3^ and the aggregate cost of healthcare and support is £27.5 billion annually^4^.

The cause of ASD is not well understood and its aetiology is complex, involving both genetic and environmental factors^5^,^6^. It is generally recognised the most effective clinical route to treatment is its early identification and consequent early therapeutic intervention^7^,^8^. Early diagnosis can also afford the family and caregivers opportunity to adjust, and in some cases can trigger the resources required for professional care and treatment. Such provision can produce significant health and economic benefit, offering the best chance for lifelong improvement and relative independence^9^,^10^.

Yet, although early diagnosis and intervention appears to offer the best chance for significant health improvement and economic gain, diagnosis of autism remains complex and often difficult to obtain. It currently relies on specialist medical expertise with diagnostic instrumentation that depends on interpretative coding of child observations, parent interviews, and manual testing. These instruments are time consuming and clinically demanding. Medical diagnosis can be withheld for many years due to wait-list times or uncertainty in clinical diagnostic fit.

Recent identification of motor disturbance in young children who develop ASD presents a new target for the development of early assessment methodologies^11^. ASD is typically considered a social and emotional disorder. Therefore, current diagnostic instruments directly address social and emotional aspects of the syndrome. However, motor control underpins social engagement, emotional expression, and cognitive development^12^,^13^,^14^,^15^,^16^,^17^, and children with ASD exhibit a clear deficit in movement observable from birth^18^ and evident throughout life^19^,^20^,^21^,^22^,^23^,^24^,^25^. This motor perspective on autism is beginning to gain some significant clinical and research interest^11^,^26^,^27^.

Disruption of normal movement patterns first identified by Kanner^28^ is a cardinal feature of ASD and is becoming increasingly recognised as a likely primary deficit in ASD aetiology^11^. Repetitive movements and restricted interests are core diagnostic criteria in professional clinical practice in both the United States (DSM-V^29^) and Europe (ICD-10^30^). However, more subtle motor disturbances, which are not included in the diagnostic classifications, are frequently observed in individuals with ASD^31^,^32^,^33^,^34^.

Individual motor kinematics of purposeful movement in tasks as varied as simple horizontal arm swings^19^, reaching to grasp^35^,^36^ or touch^37^, handwriting^38^, body posture shifts^39^, and gait^40^ are disturbed. These studies employed laboratory-based optical motion capture methods to demonstrate a reliable disruption to action kinematics in children and adults with autism. And although results may differ in terms of the precise nature of the disruption, for *e.g.* arm velocity is increased in the adult arm swing^19^, but decreased during the children’s reach^41^,^42^,^43^, all studies identify a disturbance to movement during prospective, goal-directed motor control^11^. The presentation of this disturbance may be dependent on developmental progress and on the nature of the task, as above. Some studies indicate autism motor disturbance may be coupled to intelligence^44^,^45^,^46^. Postural adjustments during load-shift tasks^39^ and during gait^23^ are also affected. And efficient prospective organisation of movements in a series, or chain, is thwarted^21^. Perceptual awareness of others’ motor intentions conveyed in body movement or eye gaze is also disrupted^47^,^48^.

A recent meta-analysis of the motor literature in autism revealed substantial motor coordination deficits pervasive across ASD diagnoses^34^. Sensorimotor timing and integration appears to be a consistent deficit, although the nature of this disruption and its effect on autism as a disorder of sensory, motor and cognitive prediction requires more work to better elucidate^11^,^33^,^49^. Nevertheless, disruptions to motor timing and coordination can thwart an individual’s intentions^14^. Such a subtle, but fundamental disruption to motor agency can create distress and isolation, and consequent autistic social and emotional compensations^11^,^50^. Prospective, feed-forward mechanisms of motor timing are a fundamental feature disrupted in autism^51^.

Thus, it would appear measures of prospective motor timing could provide a means to assess young children for autism, if such motor markers could be identified. However, research studies typically employ optical motion tracking, which is an expensive, laboratory-based system that requires expert technical operation. On the other hand, clinical assessment of motor function is typically carried out by interpreter-coded surveys, such as the M-ABC^31^ or Mullen Scales^52^, and lack precise quantification of the motor signature. More accessible and more precise computational measures of motor performance for clinical assessment and research are needed.

Recently, new technological developments have miniaturised inertial motion sensors, gyroscopes and magnetometers and integrated these into mobile consumer microelectronics. They are now ubiquitous in smart phones, tablets, and in wearable devices such as smart watches and wristbands. These new devices provide unprecedented access to motor information about the user that can be used for improved medical assessment, for example to predict Parkinson’s disease onset^53^. However, these new technologies have not yet been employed to assess motor control in children with autism.

In this study, we reasoned the new inertial sensors in smart tablet devices and touch screen sensor technologies were sufficient to capture detailed information about children’s motor patterns. The attractive nature of tablet gameplay appeared to overcome limitations of experimental motion tracking paradigms that require the subject to perform pre-set tasks wearing reflective markers inside strange, unexpected university laboratories – all under the watchful gaze of curious scientists. Such demands are difficult for individuals with autism and can affect performance, calling into question the validity of data made in these situations^54^. On the other hand, children are attracted to tablet screens and engage with them playfully of their own accord. Further, tablet devices are portable and can be brought into the home, clinic, or classroom. Altogether, development of tablet-based assessment presents an ecologically valid paradigm equipped with high precision sensors that can assess the child in attractive, paradigmatic gameplay scenarios.

We decided to test whether or not we could identify autism-specific motor patterns in the gameplay of children as they engaged with a smart tablet computer (iPad mini) under natural conditions and with minimal instructions. We reasoned this would provide more reliable information on the child’s spontaneous motor behaviour than currently available, and allow analysis of the nature of the motor disturbance by accurate measure of the child’s spontaneous, kinematic pattern of purposeful, goal-directed movements in gameplay. To do so, we adapted two commercially available games for children with code to capture the inertial sensor data and touch-screen data as the children played (Fig. 1). We then set out to computationally identify movement patterns generated by the children that reliably differentiated children with autism from typically developing children.

Thus, the aims of the study were: (1) To determine whether or not motor information could differentiate children with autism from children developing typically; and (2) to determine the kinds of movements responsible for differentiating between children with autism and children developing typically. We reasoned such identification of an ecologically valid autism motor signature and its characterisation could then be used in future research to identify younger children at risk for autism, but not yet diagnosed.

In the first game, called Sharing, the main gameplay encouraged the child to slice a piece of food by tapping on it, and then distribute the resulting pieces individually to each of four cartoon characters. On completion, the characters made a joyful exclamation before the game re-set and new piece of food was put on display for sharing. Distractor elements were also present that allowed for play outside this main task, such as turning lights on and off or tickling a bird who would then sing. In the second game, a choice of outlines of toys and animals was presented, and the selected image then placed on a clean canvas for colouring. A spinning wheel was offered with choices for colour selection, after which any touch or gesture served to colour or draw on the picture, leaving the outline always intact. This game, Creativity, allowed free play with no rules. Children were given the option to re-set the image with a new one and a clean canvas.

Altogether, 82 children were assessed: 37 children aged 4 years 5 months (standard deviation 11 months) clinically diagnosed with Childhood Autism (ICD-10 2010 Edition)^30^ were included in the autism group of the study, and 45 children 4 years 7 months (standard deviation 11 months) were included in the control group of the study. An iPad mini was placed directly in front of the children on a table so that any movement information from the device’s sensors were the result of forces made from touch, and not gross movement of the device (Fig. 2A). Two sources of information were obtained, from the touch screen and from the inertial sensors (tri-axial accelerometer, gyroscope, magnetometer) (Fig. 2B,C). 262 ‘features’ obtained by simple calculation of the raw sensor data (Supplemental Table 1) were then analysed first by machine learning algorithms in order to produce a computational model that could differentiate data patterns within ASD and Control groups. Data were then analysed by the Kolmogorov-Smirnov (KS) test. This is the first study to employ smart device serious games to study the motor patterns of children with autism spectrum disorder.

## Results

### Machine Learning Identification of an Autism-specific Action Pattern

Three machine learning algorithms were employed using 10 repetitions of a 10-fold cross-validation. Each algorithm differentiated individuals within the autism group from the control group using the 262 features derived from the touch screen and inertial sensors with accuracy up to 0.93 (Table 1). Data from Creativity gameplay produced greater predictive accuracy than those from Sharing gameplay. The most effective algorithm was the Regularized Greedy Forest^55^ with age and gender data excluded. Thus, analyses of the Creativity gameplay by RGF2 analysis produced the best predictive scores with an area under the Receiver-Operator Characteristics curve (AUC) of up to 0.93 (Fig. 3) and sensitivity and specificity up to, for example, 83% with 85% specificity (Table 2).

It is noteworthy classification of autism-specific gameplay was produced by analysis of simple computations (features) of the form and pattern of motor engagement with the device, without attention to concerns of higher cognitive function. Children with autism produced a particular motor pattern during gameplay that was significantly different from those produced by children developing typically, giving identification of a motor signature associated with childhood autism.

### The Autism Motor Signature

In order to understand the nature of this motor signature, we analysed all features using a Kolmogorov-Smirnov (KS) test. We give attention to those features with the greatest KS distance as a best approximation of those that contributed significantly to the differentiation. Thus, we focus on the ten features from each game with the greatest KS distance (Table 3; Fig. 4).

In the case of the Sharing game, all ten features were derived from simple computations of the inertial sensor data. Inertial data measure the forces resulting from impact of the finger onto the screen and therefore into the device and its sensor, as well as the forces put into the device from variances in pressure as the finger moved across it. In the case of the Sharing game, finger swipe action kinematics and form features derived from the touch screen data were not included, suggesting that a prominent component of the ASD motor signature is increased force at the point of impact.

In the case of the Creativity game, four features from the inertial sensor data and six features from the touch screen data were the most salient, indicating a significant difference both in the forces put into the device and in the action patterns of the gestures made across the screen. The latter consisted of kinematic features and measures of the final form of a gesture. Thus, both the forces put into the screen and the movement across the screen were important contributors to the autism-specific motor signature in gameplay.

Altogether, these data demonstrate the following characteristics of the autism motor signature produced in gameplay:

#### Greater Impact Force and Gesture Pressure in Autism

The inertial data indicate children with autism engaged in gameplay with greater force of impact than those developing typically. Accelerometer readings that measured impact force were higher for the autism group than controls (AccelerationRMS_y and AccelerationMagnitudeMax; Table 3; Fig. 4). Further, while the latter is a measure of absolute impact force irrespective of the vector direction, the former feature indicates greater forces along the y-axis derived from forces of the finger moving laterally.

#### Patterns of Impact Force and Gesture Pressure Differ Between Groups

Further elucidation of the impact force and gesture pressure differences were obtained from the gyroscope data. Nine of the most salient features from the Sharing game and one from the Creativity game were produced by the gyroscope (RotationCorrelation_1_2, AttitudeStdDev_y, AttitudeMean_y, RotationCorrelation_0_1, AttitudeRMS_x, AttitudeZeroCrossRate_x, RotationMean_z, RotationMeanMagnitude, RotationMin_z; Table 3; Fig. 4). Gyroscope data result from angular force vectors put into the device at the point of contact or during the gesture. Altogether, these data demonstrate children with autism applied a significantly different distribution of forces into the device during gameplay than the typically developing children did.

#### Faster Gestures in Autism

Mean gesture velocity during Creativity gameplay was greater in the autism group than in controls (Velocity; Table 3; Fig. 4).

#### Larger, More Distal Gestures in Autism

The mean area occupied by a gesture was greater in the autism group than in controls (AvgGestArea; Table 3; Fig. 4). Unexpectedly, the gestures were also located more distally in the autism group than in controls, with greater variation (GesturesHeightMax, AvgGesturesHeight, GesturesHeightStdDev; Table 3; Fig. 4).

#### Faster Screen Taps in Autism

The minimum duration of a screen tap was shorter in the autism group than in the control group (GestureDurationMin; Table 3; Fig. 4), indicating faster, rapid taps.

## Discussion

Motor patterns related to autism can be identified by machine learning from iPad gameplay in children between three and six years old. This motor signature appears to be predominantly derived from differences in pressure going into the device as well as differences in gesture kinematics and form. Importantly, of the two games, the more effective one involved only free-style colouring with no specific gameplay pattern. This confirms motor pattern, and not differences in attention to particular elements of gameplay, is a significant differentiating factor.

Nevertheless, while motor patterns appear the significant factor, other elements such as restricted attention to particular game elements may play a role in the motor patterns performed. For example, selecting and moving to different game elements may lead to different movement kinematics. This would particularly affect data that involves spatial and temporal gestural aspects, i.e. touch screen features. However, such behavioural differences are unlikely to affect the force related parameters that result from regulation of gesture contact, i.e inertial sensor features. Importantly, it is these latter features that provided the majority of the most salient features for differentiation between groups, supporting the notion fundamental disruption to motor pattern is the more important differentiator.

Further, these data reveal new insight into the nature of the motor disturbance. Forces put into the device on contact differentiated between children with ASD and typically developing ones. The children with autism displayed greater force at impact and a different pattern of force put into the device during gestures than their typically developing peers. This is likely caused by maintaining greater velocity at contact with consequent increased impact force, supporting the notion that prospective guidance of goal-directed movement is disrupted in ASD^11^, causing over- and under-compensations over the course of a movement, for *e.g.* reaching to touch the screen or moving the finger across the screen.

This finding is in line with results from optical motion tracking experiments of goal-directed tasks, which demonstrate individuals with autism make greater subsecond moment-by-moment adjustments to the progression of a movement toward its goal than neurotypical individuals do^19^,^24^,^56^. For example, in a simple arm swing task, the amplitude of the jerk (rate of change of acceleration) was significantly greater in autism, as was the swing peak velocity^19^. Similarly, in a grasp-and-place task, children with ASD made multiple corrective movements over its course and had higher velocities at the movement terminus^57^. The grasp itself requires precise maintenance of force into the object, with enough force to maintain the grasp and without under- or over-exertion. Reach-to-grasp kinematics and grasp force patterns are significantly different in autism^45^, with greater grasp force and more variable performance^58^. Continuous and regular under- and over-exertion of movement in autism is evident. In our paradigm, these create differences in the magnitude and variance of force put into the device, which are recorded as acceleration and rotation signals. We provide a novel means and identify for the first time the significance of contact force as an important feature of the autism motor signature.

These findings support the notion of a core deficit in autism of the prospective control of movement^11^, or motor agency^14^, evident in a growing body of data on disruption to anticipatory, or feed-forward mechanism of goal-directed action in autism^44^,^45^,^59^. Further, disruption to perception of others’ motor goals and their affective salience (vitality form) is also disrupted in autism^47^,^48^,^60^, suggesting a core deficit in autism in sensorimotor timing and integration. Visuomotor resonance that affords perception-action coupling, as well as social understanding of the motor intentions of others may be disturbed^61^.

Such disruptions may have an aetiological root in basic brainstem sensorimotor information processing^11^ affecting the consequent dynamic of social engagement from birth^11^,^50^,^62^. Brainstem growth is affected in autism^63^,^64^,^65^, and these disruptions likely give rise to structural and functional errors, especially of the inferior olive responsible for the fast, subsecond control of skilled movement^66^. Evidence indicates brainstem white matter tract connectivity associates with autism severity and motor control efficiency^67^. Downstream developmental consequences of an early brainstem disruption may lead to autistic socio-emotional and cognitive compensations^11^,^68^. Alternatively, proprioceptive feedback that allows online guidance of movement may be disrupted, creating resonance and control errors^24^. While the neurobiological source of the disruption requires more work to resolve, the particular autism motor signature appears in our data here to be a sensitive marker for children with the disorder.

The motor disturbance is first evident at birth by retrospective video analysis^18^, but its prognostic, or diagnostic value has not yet been realised. Atypical object exploration by young infants who are at-risk of ASD or later diagnosed with ASD appears to support a motor deficit model^69^,^70^. And retrospective parent reports on oral-motor behaviour do predict autism^71^, but measures of motor performance in infancy for prospective prediction of autism have not been forthcoming. Similarly, the pattern of social eye gaze in early infancy can be significantly different for those who develop autism^72^, but measurement of these differences is not sufficient to predict autism and early behavioural markers remain elusive.

Thus, although some considerable effort has been made toward discovery of biomarkers, these have not been forthcoming. The heterogeneity of the disorder and its complex aetiology have meant standard biological indicators have proved elusive^5^,^6^,^73^. Given its complex aetiological picture, we propose the search for markers as purely biological entities (genes, molecules, metabolites, neuroanatomy) may be misplaced and that, instead, computational bio-behavioural markers, such as those we have identified here, may prove more effective in robust, early identification of autism than traditional methods.

To this end, this study presents new methodology for the computational identification of autism. But in order to realise the goal of a computational bio-behavioural marker for autism, the specificity of this motor signature requires further testing to eliminate potential confounders. The motor signature we identify here may overlap with other developmental disorders, such as developmental coordination disorder or attention deficit hyperactivity disorder. These disorders have some common motor features with autism^31^,^74^,^75^, although they also exhibit distinct differences^76^. Further study is needed to resolve the detail of these patterns.

An ambition of this work is to develop an accessible, attractive serious gameplay paradigm that can be commercialised as an economic, labour-free addition to the current diagnostic toolbox, or as a screening device for health and educational services, or concerned parents. The present study is a first proof-of-concept in this development. The paradigm employed here tests children already diagnosed with ASD against children with no concern for ASD. Although this is not the ultimate clinical question, because children with typical development are rarely a cause for concern and are therefore rarely referred to clinics for evaluation, it provides an essential first step in this direction. The ultimate clinical question is whether or not motor patterns can differentiate ASD from other disorders that are not ASD, *i.e.* between two children both exhibiting symptoms that could be perceived as ASD-like. This question – sensitive differentiation between ASD and ASD-like clinical presentation – will the subject of future study.

In terms of limitations of the present study, we were unable to exclude intelligence as a potential confounder, since our groups were not controlled by intelligence quotient. However, most of the children who participated in the study were classified by their clinicians as average (N = 29) and high functioning (N = 4). Only four children were classified as low functioning, of which only two finished the study and contributed to the final data. For this reason, the potential for a confounding effect by differences in intelligence between groups is diminished.

Further, our study design employed subject recruitment from particular institution, giving this study a picture of the performance profile of a subgroup of children on the autism spectrum. Thus, this proof-of-concept study does not necessarily reflect the full population-wide variance in autism spectrum disorder gameplay patterns, but may be specific to those kinds of children recruited into those clinics. This inherent selection bias in our autism group may have facilitated machine learning differential power. Future work is required to test whether these algorithms remain predictive for the general population, or if they require re-training. Finally, machine learning algorithms are sensitive to the sample size. Thus, future studies to establish a generalized serious game assessment of autism need to include wider recruitment parameters and larger numbers.

Only one other study has successfully employed machine learning for identification of autism-specific motor patterns, by analysis of optical motion capture data of children in a reach-grasp-place paradigm^42^. This study achieved an accuracy of 96%, but employed a lower sample size (N = 30), Support Vector Machine approach, and a leave-one-out cross-validation that altogether render this methodology prone to over-fitting. In our study, we worked to reduce over-fitting with larger subject numbers (N = 82), a Regularized Greedy Forest approach, and 10 repetitions of a 10-fold cross-validation procedure. Nevertheless, the fact that two different paradigms (experimental and serious game) employing two different data capture methodologies (optical and smart device) achieved similar result through machine learning demonstrates the significance of motor measures as a likely best possible target for an early bio-behavioural marker of autism.

In conclusion, we have shown here that smart tablet technology offers an attractive, new paradigm for clinical autism assessment and bio-behavioural research of pre-school children, enabling engaging, ecological testing of children’s motor behaviour in a fun, accessible format fit for precise computational analysis of neuropsychological function. Further development of this smart gaming paradigm can ultimately lead to improved functional assessment of a child’s particular individual characteristics, rather than categorisation by neuropsychiatric systems of diagnosis that may be too broad for clinical or therapeutic utility^77^. The technology has the potential to be coupled to other psychometric tests adapted into bespoke gameplay, and may employ the sensors in novel ways, such as tests of social intelligence or emotional reaction detected by the face-forward camera, or by coupling in gameplay with sensorised toys^78^,^79^.

In sum, we show that children with autism can be identified with up to 93% accuracy by computational analysis of their motor pattern in iPad gameplay. This differential power is based on simple computations of the device’s sensor data (inertial sensors and touch screen) that altogether describe an individual’s motor signature as they touch, tap, swipe, or move the finger across the device. Disturbance of gesture force patterns appear significant contributors to the autism motor signature and provide new insight into the nature of the motor deficit as a disorder of prospective, anticipatory motor guidance. These data support the notion that motor differences are a significant, possibly a core component of autism spectrum disorder expression. Future work is now required to substantiate this first study with a larger, more generalised population and to test the algorithms for strength of differentiation between ASD and other development psychopathologies. Improved knowledge of the autism motor signature can then be applied to younger children, and those children who do not yet have a diagnosis of autism. In sum, machine learning identification of autism spectrum disorder by motor analysis of serious tablet gameplay appears a promising new methodology for early detection of autism, enabling computational assessment of a putative bio-behavioural marker in an enjoyable, ecological, accessible serious game paradigm.

## Method

### Participants

37 children aged 3 to 6 years old (mean 4 years 5 months, standard deviation 11 months) clinically diagnosed with Childhood Autism (ICD-10 2010 Edition; World Health Organisation, 2011) were included in the ‘Autism’ group of the study. Of these, 12 were female. 45 children age-matched (mean 4 years 7 months, standard deviation 11 months) and gender matched (13 female) typically developing children were included in the ‘Control’ group of the study. All participants had normal or corrected-to-normal vision and no other sensory or motor deficits. Those children who could not follow simple instruction were excluded.

Children in the ‘Autism’ group were recruited at specialist therapeutic centres and selected by clinicians. Diagnosis was obtained by medical practitioners working within the specialist clinics. Of the 37 children diagnosed with Childhood Autism, 30 were uncomplicated, one was considered ‘high functioning’, two were diagnosed with Asperger’s Syndrome, and four were considered co-morbid with ‘intellectual impairment’, of which two completed the study and contributed to the final data. Children in the ‘Control’ group were recruited at standard kindergartens.

A questionnaire regarding severity of autistic symptoms, level of intellectual and social functioning, and experience using mobile devices was carried out with the clinicians in the case of the ‘Autism’ group, and with teachers in the case of the ‘Control’ group. Any child whose clinician or teacher was uncertain about the child’s diagnosis or health was excluded.

Prior to the study, children’s parents gave written informed consent for their children’s participation. The experimental protocols employed were carried in out in accordance with the Declaration of Helsinki and approved by the University of Strathclyde Ethics Committee.

### Materials

The study was performed on iPad mini tablet computers (Apple Inc.) running standard iOS version 7.0. Two educational games designed by Duckie Deck Game Studio (www.duckiedeck.com) for children aged 2–5 years and commercially available were employed. These games were presented to the children within a bespoke app that organised the display of the games sequentially and with fixed time periods for each game. The app also locked the device into the game for the duration of the experiment, disabling the ‘home’ button. It included code for collecting the sensor and touch screen data (described below).

**Figure 1:**
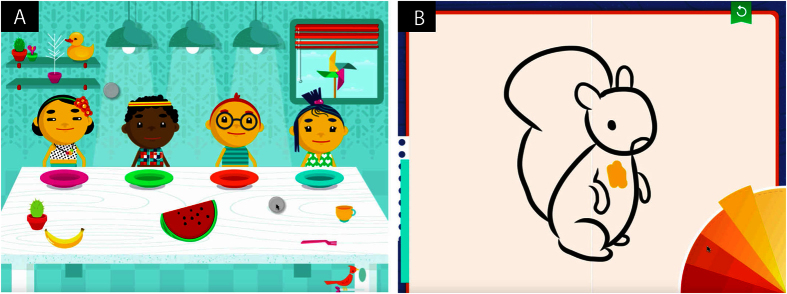
The two serious tablet games employed for data capture. (**A**) ‘Sharing’ where the main gameplay involved touching the fruit (centre forward), which sliced it into four equal pieces, then sliding each piece to a child’s plate. When all four children had a slice of fruit, they would jump for joy for 3 seconds before the fruit was replaced with another food, and the children would return to their neutral position. (**B**) ‘Creativity’ where the children were free to choose an object or animal shape, then trace the shape before colouring it in freely, choosing a colour from the colour wheel. When the children were satisfied, they could choose a new shape by selecting the return button in the top right-hand corner.

The two games were attractive and fun for children and engaged their gameplay without verbal instruction (Fig. 1). Animated cartoon characters responded to the child’s interactive gestures with simple changes of facial, gestural, and non-verbal vocal expression. Toys and objects within the gameplay environment were responsive to touch gestures and were either included in the main gameplay with responses from the principle characters, or were considered ‘distractors’ with only a localised response that did not affect the other gameplay elements, for *e.g.* birds, window shades, and light bulbs that responded to touch with a song, change of scene, or illumination, together with appropriate and playful sounds. The two games were as follows:

#### Sharing

The main gameplay consisted of dividing a piece of food, for *e.g.* an apple, and distributing it evenly among four children present on the screen. The game consisted of a series of these trials. The child’s task was identical in each trial, however the food object, which the child divided and shared, differed from trial to trial. Gameplay was simple and suitable for children 3–6 years old. The food was divided into four even portions with a simple touch, each piece could then be dragged and dropped onto the plate in front of each cartoon child, who gave a positive facial expression when the user did so. When the food was distributed evenly, all children exclaimed, “Yipee!” and proceeded to munch the food in a delightful manner for 3 seconds. Then the trial repeated. If food was distributed unevenly, the children with a piece of food or a pile of pieces of food remained with a positive expression, but the children with empty plates frowned with a negative vocalisation, “Auh.” In this way children could play with dividing and sharing the food, to the delight or frustration of the characters. In addition, the game included a number of ‘distractor’ objects whose role was not involved in the main gameplay, but nevertheless presented enjoyable visual and acoustic responses.

#### Creativity

This game was open with minimal structure. It did not involve any specific ‘rules’ of engagement with objects and characters. Instead, the game involved a single round of outlining, then colouring a picture of a toy or animal. First, the child was asked to choose a shape from a set of objects on a scrolling slider, for *e.g.* a squirrel, a robot, a flower. A dotted outline of each element of the drawing then appeared, and a simple swipe near the outline filled in the line fully. This interactive pattern was repeated through each outline element until the drawing was complete. A colouring wheel then appeared and spun in the bottom right-hand corner of the screen, indicating a choice for selection. The child could then select a colour and any subsequent screen touch would paint that colour onto the picture. The toy or animal outline always remained unobstructed, but the child could paint and colour anywhere freely. A small button on the top right-hand side allowed the child to refresh the colouring page by selecting a new toy or animal. Thus, the user was enabled to engage in creative drawing and colouring at liberty until the game time was up.

### Experimental Gameplay Protocol

**Figure 2:**
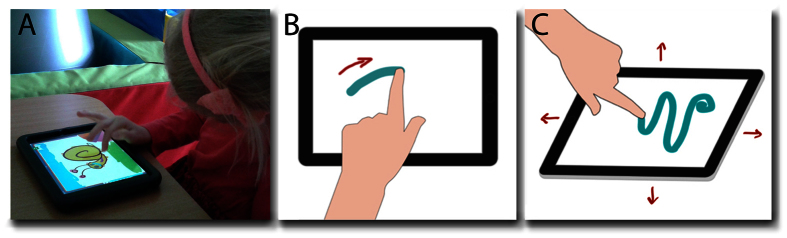
The child’s purposeful movements were sensed by the touch screen and the inertial sensors inside the tablet.

Each participant was seated at a children’s table *ca.* 65 cm high and asked to play the games on an iPad mini placed on the table directly in front of him or her within 10 cm from the table’s edge (Fig. 2A). Each game consisted of a 2 minute training phase followed immediately by a single 5 minute test phase. The training phase included a series of guides that encouraged attention to the main gameplay using arrows and animation to demonstrate the gameplay patterns, for *e.g.* in Sharing the food would jump up and down until the child touched it, then arrows would indicate sliding the pieces to each child. In this way, the child engaged playfully to learn the principal game patterns and responses. Each training phase and test phase ended automatically after the time elapsed.

At the beginning of the training, an experimenter explained the aim of the game to a child verbally and using gestures on the game. The child was supported during this phase. In the test phase, the experimenter no longer interfered with the child’s gameplay. The child’s clinician or teacher was present during the experimental gameplay, and intervened only when challenging behaviours occurred. In these cases the data were omitted from the study.

All typically developing children and 35 of 37 of children with autism engaged in gameplay. 2 participants from in the Autism group did not focus on the application or resigned from gameplay during the experiment and their data were discarded.

### Data acquisition and pre-processing

Tablet gameplay enabled four different kinds of interaction: a ‘tap’ with a single digit, a simultaneous ‘multi-touch’ with two or more digits, a ‘touch movement’ across the screen once one or more finger were touching the screen, and finally the release of the digit or digits from the screen. For the sake of clarity, we name all classes of interaction, ‘gestures’. Touch data from gestures across the screen (Screen) and touch data from tablet’s inertial movement sensors (Inertial) (tri-axial gyroscope and tri-axial accelerometer) were collected (Fig. 2B,C). For acquisition of touch data, which included information about single-and-multi touch events, standard acquisition methods embedded in the iOS system were used. Data from sensors were acquired using iOS Core Motion framework at a rate of 10 Hz. Subsequently, data were transferred to cloud services via web, using small compressed (gzip) packages with JavaScript Object Notation (JSON) data. In the cloud data were divided into types: *Touch Data* pertaining to the way in which a child made the gestures on the screen and *Sensor Data* obtained from the accelerometer and gyroscope. These data were stored in tables using NoSQL Microsoft Azure Table Storage technology. The iPad tablets were used only for data acquisition and temporary storage, with all subsequent data handling and computation performed within cloud services.

### Feature extraction and selection

Two hundred sixty-two features were extracted from these data to give a comprehensive computational description of the child’s movements sensed by the device, and made in interaction with it (Supplemental Table 1). These features were obtained from the Screen (108 features) and Inertial (164 features) data. Of these features, 26 were highly correlated (r > 0.9), considered redundant, and reduced to a single feature. 247 features from device sensors and touch, together with information about child’s age and gender, were included in the final analysis.

Features were computed from consecutive sets of raw data using a dedicated, bespoke engine. Touch and inertial sensor features were calculated for each gaming session. Touch data for each gaming session were aggregated and split into atomic gestures based on the start and end of any particular gestures. For every gesture, sets of variables were calculated. These features can be split into two major groups: (i) features of movements’ kinematics, for *e.g.* velocity and acceleration, and tap-based features, for *e.g.* the number of taps in a game. Inertial sensor values were computed across the game session irrespective of the touch data. The values for each feature for each game were then reduced to its mean and used as input for machine learning.

### Machine Learning Data Analysis

The machine learning approach is shown diagrammatically in Fig. 5. Data were labelled accordingly to the child’s diagnostic group (Autism or Control) and age. Touch and inertial sensor features as well as labels were fed into machine learning algorithms. The models (described below) estimated the probability that a particular child’s data belonged to one group or the other.

**Figure 3:**
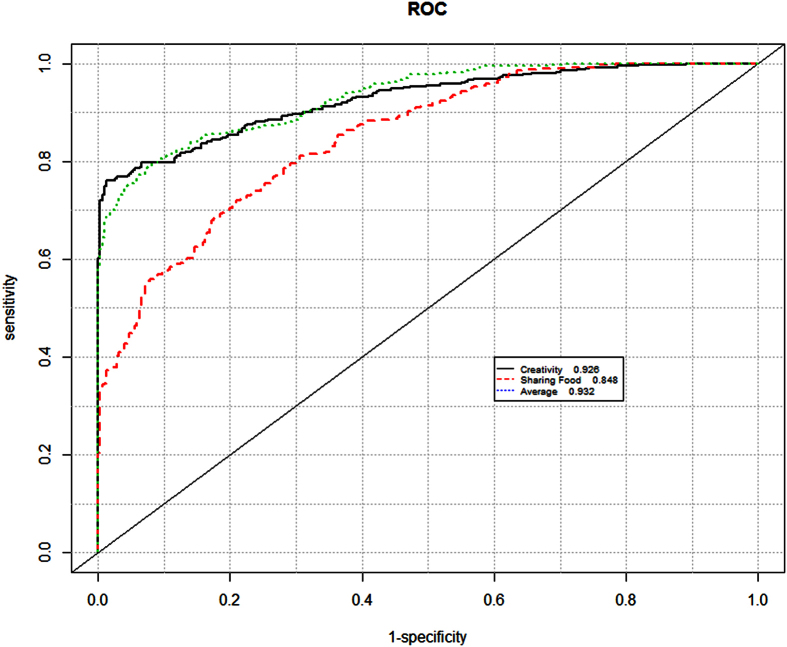
Receiver operating characteristic curves (ROC) of the RGF2 models. For higher classification thresholds (moving to the left on the plot; higher specificity, lower sensitivity) Creativity is the best performer. The plot was obtained by aggregating all predictions from 10 repetitions of 10-fold cross-validation (740 observations).

**Figure 4:**
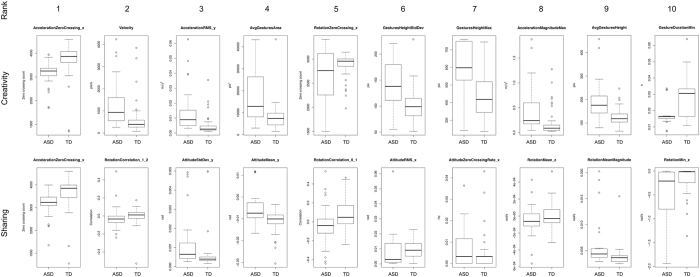
Boxplots of the ten features with the greatest Kolmogorov-Smirnov distance between Autism and Control groups for the Creativity and Sharing games. Descriptions of these features are given in Table 3. Boxplots show median values (horizontal line), interquartile range (box outline), minimum and maximum values of the upper and lower quartiles (whiskers) and outliers (circles).

**Figure 5:**
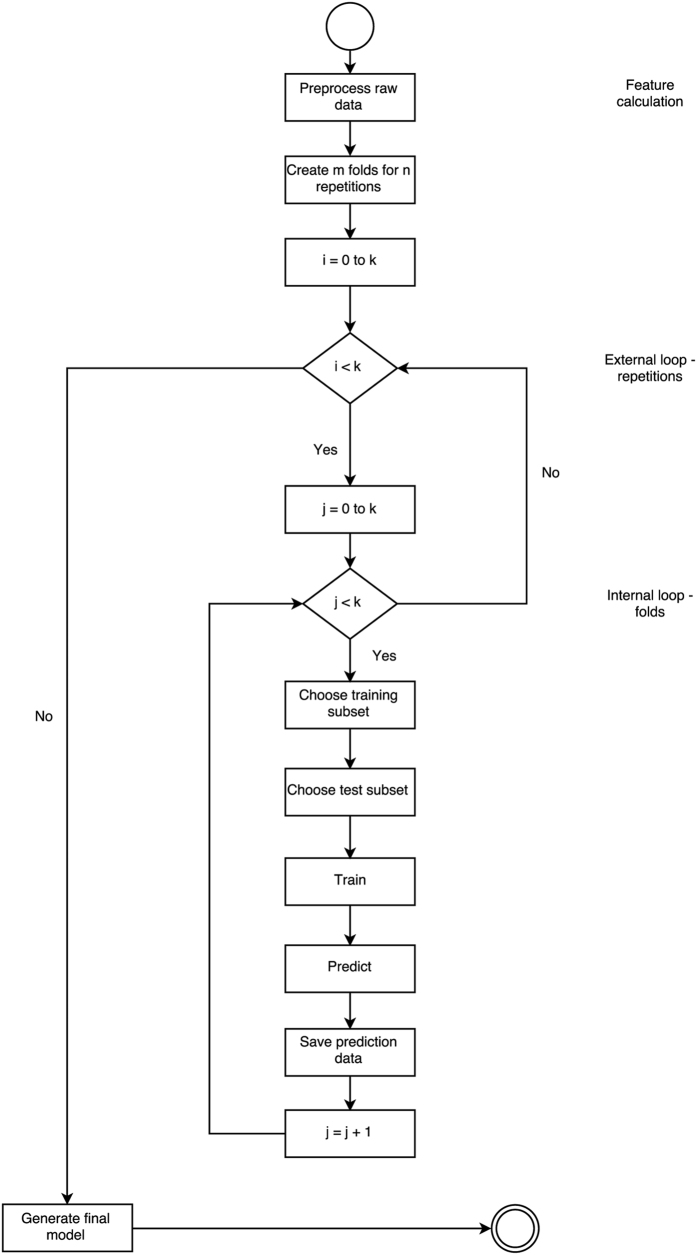
The machine learning approach.

To build models able to predict group classification (ASD or Control), and to ensure reliable classification, a *k*-fold cross validation procedure was employed. This method was used to establish the predictive power of the model, *i.e.* how the result ought to generalise to an independent dataset. To increase the stability of the result, additional k repetitions of the process were performed. The full dataset of calculated features was split into *k* equal sized samples. From *k* subsamples one was chosen for the validation (test), and the rest (*k* −1) were used as the training dataset. Every sample was used exactly once as a validation (test) dataset. This process was performed *k* times (folds). During every iteration model was trained on the *k*-1 sample and then tested on the one sample left for the prediction. Results of the prediction were stored, to later establish the end-point model, which combines the results from each fold. This process was repeated *k* times. In the end *k* × *k* samples were created and tested. Based on the prediction data gathered during both iterations (repetitions and folds), the Receiver Operating Characteristics (ROC) curve (Fig. 3) was generated, and the sensitivity (true positive rate) and specificity (true negative rate) (Table 2) were calculated.

In this study, a 10-fold cross-validation procedure was performed on the data from each game. Furthermore, 10 repetitions of the procedure were made to ensure stability of result. Area under the receiver operating characteristic curve (AUC) was calculated. This measure does not require selection of a fixed classification threshold, *i.e.*, it is able to simultaneously investigate performance over a range of thresholds [0, 1]. An AUC of 0.5 means a random classification and 1 indicates perfectly separated classes.

Algorithms that could be prone to overfitting on such a small dataset, like Gradient Boosting Machines or Support Vector Machines, were not used in the analysis. Taking into account that there were significantly more features than observations, and that many high (r > 0.9) linear correlations between features were found the main focus was put on decision-tree-based ensembles. Several algorithms were evaluated after appropriate parameter searches, namely ExtraTrees (ET)^80^, Random Forest (RF)^81^, and Regularized Greedy Forest (RGF)^55^. Additionally, a second run of the RGF was employed with information about child’s age and gender excluded from the analysis, RGF2. In all other cases, information about child’s age and gender were included as features in the analysis.

Finally, an approximation of the features that were most effective in differentiating ASD patterns from TD ones was performed using the Kolmogorov-Smirnov (KS) test. Those with the greatest KS distance between groups were examined.

## Additional Information

**How to cite this article**: Anzulewicz, A. *et al.* Toward the Autism Motor Signature: Gesture patterns during smart tablet gameplay identify children with autism. *Sci. Rep.* **6**, 31107; doi: 10.1038/srep31107 (2016).

## Supplementary Material

Supplementary Information

## Figures and Tables

**Table 1:** AUC mean (and standard deviation) determined by 10 repetitions of 10-fold cross-validation.

Algorithm	Sharing Food	Creativity	Average
ET (5000 trees)	0.785 (σ = 0.016)	0.893 (σ = 0.01)	0.881 (σ = 0.01)
RF (5000 trees)	0.802 (σ = 0.017)	0.892 (σ = 0.006)	0.885 (σ = 0.006)
RGF (500 trees, L2 = sL2 = 1.0, square loss)	0.835 (σ = 0.017)	0.921 (σ = 0.012)	0.927 (σ = 0.011)
RGF2	0.848 (σ = 0.025)	0.926 (σ = 0.013)	0.932 (σ = 0.016)

The last column (Average) denotes AUC obtained by taking a mean of predictions of both games for each child.

**Table 2:** Sensitivity and specificity of RGF2 for the Sharing Food and Creativity games with thresholds selected at 0.50 and 0.55 to show the performance of models more intuitively.

	Sensitivity [%]	Specificity [%]
Sharing Food (0.50)	0.81	0.67
Sharing Food (0.55)	0.76	0.73
Creativity (0.50)	0.83	0.85
Creativity (0.55)	0.80	0.88

Selecting a lower threshold (here 0.5) corresponds to moving to the right on the ROC curve, thus raising sensitivity, while decreasing specificity.

**Table 3:** Features with the greatest Kolmogorov-Smirnov distance between Autism and Control groups for the Creativity and Sharing games.

KS distance ranking	Inertial (I) or Touch (T)	Feature name	Description
Creativity			
1	I	AccelZeroCrossing_x	Accelerometer x-axis (longitudinal) value sign (+/−) change count.
2	T	Velocity	Mean gesture velocity.
3	I	Accel RMS_y	Root mean square of accelerometer y-axis (lateral) values.
4	T	AvgGestArea	Mean area occupied by a gesture, computed as the area occupied by a minimal adaptive polygon fitted to the gesture.
5	I	RotationZeroCrossing_z	Gyroscope z-axis (vertical) value sign (+/−) change count.
6	T	GesturesHeightStdDev	Standard deviation of height (x-axis in landscape) values.
7	T	GesturesHeightMax	Maximum value of height (x-axis in landscape).
8	I	AccelerationMagnitudeMax	Maximum accelerometer value irrespective of axis.
9	I	AvgGesturesHeight	Mean height (x-axis in landscape) value.
10	T	GestureDurationMin	Minimum duration of a touch gesture.
Sharing			
1	I	AccelZeroCrossing_x	Accelerometer x-axis (longitudinal) value sign (+/−) change count.
2	I	RotationCorrelation_1_2	Pearson product-moment correlation coefficient between gyroscope y- and z-axis rotation values.
3	I	AttitudeStdDev_y	Standard deviation of the gyroscope static y-axis values.
4	I	AttitudeMean_y	Mean of the gyroscope static y-axis values.
5	I	RotationCorrelation_0_1	Pearson product-moment correlation coefficient between gyroscope x- and y-axis rotation values.
6	I	AttitudeRMS_x	Room mean square of the gyroscope static x-axis values.
7	I	AttitudeZeroCrossRate_x	Frequency of the sign (+/−) change of gyroscope x-axis.
8	I	RotationMean_z	Mean value of the gyroscope z-axis rotation.
9	I	RotationMeanMagnitude	Mean value of the norm of the gyroscope rotation.
10	I	RotationMin_z	Minimum value of the gyroscope z-axis rotation.
