# Supplementary material for: Toward the Autism Motor Signature: Gesture patterns during smart tablet gameplay identify children with autism
Source: Sci Rep. 2016 Aug 24;6:31107. doi: 10.1038/srep31107 (PMC4995518; doi:10.1038/srep31107)
Supplement: Supplementary Information [file srep31107-s1.pdf]

Anzulewicz, A., Sobota, K. & Delafield-Butt, J. T. Toward the autism motor signature: Gesture patterns during smart tablet gameplay identify children with autism. *Sci. Rep.* **6**, doi:10.1038/srep31107 (2016).

Supplemental Table 1. Simple computational features derived from the iPad's sensors and selected for machine learning analysis.

Feature Name
**Touch Screen Features**
Velocity
Acceleration
Deceleration
MovementAngle
DirectDistance
MovementEfficiency
MovementUniformity
MovementTargeting
Skewness
MoveAndTapDelay
TapPrecision
MovementSpeed
TapFrequency
Jerk
OvershotNumber
OvershotLength
TouchGestureCount
AvgDirectnessIndex
DirectnessIndexMin
DirectnessIndexMax
DirectnessIndexRange
DirectnessIndexStdDev
DirectnessIndexMedian
GestureDurationMedian
GestureDurationMin
GestureDurationMax
GestureDurationRange
GestureDurationStdDev
AvgGestureDuration
GesturesAreaMedian

GesturesAreaMin
GesturesAreaMax
GesturesAreaRange
GesturesAreaStdDev
AvgGesturesArea
GesturesHeightMedian
GesturesHeightMin
GesturesHeightMax
GesturesHeightRange
GesturesHeightStdDev
AvgGesturesHeight
GesturesWidthMedian
GesturesWidthMin
GesturesWidthMax
GesturesWidthRange
GesturesWidthStdDev
AvgGesturesWidth
GestureVelocityMedian
GestureVelocityMin
GestureVelocityMax
GestureVelocityRange
GestureVelocityStdDev
AvgGestureVelocity
TouchEventsPerGestureMedian
TouchEventsPerGestureMin
TouchEventsPerGestureMax
TouchEventsPerGestureRange
TouchEventsPerGestureStdDev
AvgTouchEventsPerGesture
GesturesAccelerationMedian
GesturesAccelerationMin
GesturesAccelerationMax
GesturesAccelerationRange
GesturesAccelerationStdDev
AvgGesturesAcceleration
GesturesDecelerationMedian
GesturesDecelerationMin
GesturesDecelerationMax
GesturesDecelerationRange
GesturesDecelerationStdDev
MultitouchGesturesCountMedian
MultitouchGesturesCountMin
MultitouchGesturesCountMax
MultitouchGesturesCountRange

MultitouchGesturesCountStdDev
AvgMultitouchGesturesCount
TapNumberMedian
TapNumberMin
TapNumberMax
TapNumberRange
TapNumberStdDev
AvgTapNumber
MovementBreaksMedian
MovementBreaksMax
MovementBreaksRange
MovementBreaksStdDev
AvgMovementBreaks
AvgGesturesJerk
GesturesJerkMin
GesturesJerkMax
GesturesJerkRange
GesturesJerkMedian
GesturesJerkStdDev
**Inertial Sensor Features**
AccelerationMean_x
AccelerationMean_y
AccelerationMean_z
AccelerationCorrelation_0_0
AccelerationCorrelation_0_1
AccelerationCorrelation_0_2
AccelerationCorrelation_1_0
AccelerationCorrelation_1_1
AccelerationCorrelation_1_2
AccelerationCorrelation_2_0
AccelerationCorrelation_2_1
AccelerationCorrelation_2_2
AccelerationMagnitudeMax
AccelerationMagnitudeMin
AccelerationMax_x
AccelerationMax_y
AccelerationMax_z
AccelerationMeanMagnitude
AccelerationMedian_x
AccelerationMedian_y
AccelerationMedian_z
AccelerationMin_x
AccelerationMin_y

AccelerationMin_z
AccelerationRange_x
AccelerationRange_y
AccelerationRange_z
AccelerationRMS_x
AccelerationRMS_y
AccelerationRMS_z
AccelerationStdDev_x
AccelerationStdDev_y
AccelerationStdDev_z
AccelerationZeroCrossing_x
AccelerationZeroCrossing_y
AccelerationZeroCrossing_z
AccelerationZeroCrossingRate_x
AccelerationZeroCrossingRate_y
AccelerationZeroCrossingRate_z
JerkMean_x
JerkMean_y
JerkMean_z
JerkCorrelation_0_0
JerkCorrelation_0_1
JerkCorrelation_0_2
JerkCorrelation_1_0
JerkCorrelation_1_1
JerkCorrelation_1_2
JerkCorrelation_2_0
JerkCorrelation_2_1
JerkCorrelation_2_2
JerkMagnitudeMax
JerkMagnitudeMin
JerkMax_x
JerkMax_y
JerkMax_z
JerkMin_x
JerkMin_y
JerkMin_z
JerkMedian_x
JerkMedian_y
JerkMedian_z
JerkMeanMagnitude
JerkRange_x
JerkRange_y
JerkRange_z
JerkRMS_x

JerkRMS_y
JerkRMS_z
JerkStdDev_x
JerkStdDev_y
JerkStdDev_z
JerkZeroCrossing_x
JerkZeroCrossing_y
JerkZeroCrossing_z
AttitudeMean_x
AttitudeMean_y
AttitudeMean_z
AttitudeCorrelation_0_0
AttitudeCorrelation_0_1
AttitudeCorrelation_0_2
AttitudeCorrelation_1_0
AttitudeCorrelation_1_1
AttitudeCorrelation_1_2
AttitudeCorrelation_2_0
AttitudeCorrelation_2_1
AttitudeCorrelation_2_2
AttitudeMagnitudeMax
AttitudeMagnitudeMin
AttitudeMax_x
AttitudeMax_y
AttitudeMax_z
AttitudeMeanMagnitude
AttitudeMedian_x
AttitudeMedian_y
AttitudeMedian_z
AttitudeMin_x
AttitudeMin_y
AttitudeMin_z
AttitudeRange_x
AttitudeRange_y
AttitudeRange_z
AttitudeRMS_x
AttitudeRMS_y
AttitudeRMS_z
AttitudeStdDev_x
AttitudeStdDev_y
AttitudeStdDev_z
AttitudeZeroCrossing_x
AttitudeZeroCrossing_y
AttitudeZeroCrossing_z

AttitudeZeroCrossingRate_x
AttitudeZeroCrossingRate_y
AttitudeZeroCrossingRate_z
RotationMean_x
RotationMean_y
RotationMean_z
RotationCorrelation_0_0
RotationCorrelation_0_1
RotationCorrelation_0_2
RotationCorrelation_1_0
RotationCorrelation_1_1
RotationCorrelation_1_2
RotationCorrelation_2_0
RotationCorrelation_2_1
RotationCorrelation_2_2
RotationMagnitudeMax
RotationMagnitudeMin
RotationMax_x
RotationMax_y
RotationMax_z
RotationMeanMagnitude
RotationMedian_x
RotationMedian_y
RotationMedian_z
RotationMin_x
RotationMin_y
RotationMin_z
RotationRange_x
RotationRange_y
RotationRange_z
RotationRMS_x
RotationRMS_y
RotationRMS_z
RotationStdDev_x
RotationStdDev_y
RotationStdDev_z
RotationZeroCrossing_x
RotationZeroCrossing_y
RotationZeroCrossing_z
RotationZeroCrossingRate_x
RotationZeroCrossingRate_y
RotationZeroCrossingRate_z

Notes:

Touch Features:

[FeatureName][Suffix(optional)]

Every feature starts with the name e.g. Velocity

Some of the features contains also type e.g. GesturesHeight**Median** indicates median of the gestures height

Sensors Features:

[Data Type][FeatureName][Suffix(optional)]

Each feature name starts with the data type used to calculate feature: Acceleration for accelerometer based features, Rotation for raw gyroscope data, Attitude for Euler angles calculated from the gyroscope data

Second factor is the feature name

There’re two type of suffixes:

_x,_y,_z for the position in 3D space(vector)

_number_number (e.g. _1_1) for matrices. First number indicates row, second column in the matrix
